# Nanozymes for Energy and Environmental Sustainability

**DOI:** 10.1002/advs.202519402

**Published:** 2026-01-28

**Authors:** Xiaoqi Li, Jinxing Chen, Shaojun Dong

**Affiliations:** ^1^ State Key Laboratory of Electroanalytical Chemistry, Changchun Institute of Applied Chemistry Chinese Academy of Sciences Changchun China; ^2^ School of Applied Chemistry and Engineering University of Science and Technology of China Hefei China

**Keywords:** energy conversion, environmental remediation, hydrolases, nanozyme, oxidoreductases

## Abstract

Nanozymes have shown remarkable promise in addressing pressing challenges in energy and environmental sustainability. Their enzyme‐like catalytic activity, combined with exceptional chemical stability, low cost, and structural tunability, enables them to function effectively in harsh operational conditions where natural enzymes typically fail. In environmental applications, nanozymes have been employed for pollutant degradation, wastewater treatment, heavy metal detoxification, and antibacterial disinfection, offering high efficiency, reusability, and adaptability across different media. In the energy sector, nanozymes contribute to critical reactions such as oxygen reduction, hydrogen evolution, and carbon dioxide conversion, supporting the development of biofuel cells, artificial photosynthesis systems, and electrocatalytic processes. This review highlights recent advances in nanozyme design for energy and environmental applications, emphasizing their functional advantages, underlying mechanisms, and integration into scalable, green technologies.

## Graphical abstract

At present, the widely used nanozymes are oxidoreductase and hydrolase, which have proved to have great application prospects in energy and environment.

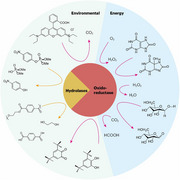

## 1 Introduction

The increasing escalating global challenges of climate change, energy scarcity, and environmental pollution have reinforced the urgent need for sustainable catalytic technologies [1]. Conventional catalytic systems, including both homogeneous and heterogeneous catalysts, have contributed substantially to energy conversion and environmental remediation [2, 3]. Nevertheless, their widespread deployment is frequently constrained by intrinsic drawbacks such as high cost, limited operational stability, and inadequate selectivity under complex or harsh conditions [4]. In contrast, natural enzymes display exceptional catalytic efficiency and substrate specificity in mild environments [5, 6]. However, their practical application is hindered by instability, high production costs, and susceptibility to denaturation in nonphysiological settings [7].

In light of these challenges, nanozymes—nanomaterials with intrinsic enzyme‐like catalytic activities—have emerged as a transformative class of artificial catalysts that bridge the gap between natural biocatalysts and conventional inorganic systems [8]. Since their discovery, the field of nanozymes has expanded rapidly to encompass a broad spectrum of materials, including metal and metal oxide nanoparticles, carbon‐based nanostructures, single‐atom catalysts and metal–organic frameworks [9, 10, 11, 12, 13, 14]. Notably, these materials mimic a wide range of enzymatic functions, particularly those of oxidoreductases (for example, peroxidase, oxidase, and catalase) as well as hydrolases, while offering several critical advantages, such as robust physicochemical stability, low fabrication cost, tunable surface chemistry, and resilience under harsh operational conditions. The distinctive properties of nanozymes have facilitated their integration into a broad spectrum of sustainability‐oriented technologies. In the field of environmental remediation [15], they have demonstrated considerable efficiency in the degradation of organic pollutants [16], heavy‐metal detoxification [17], microbial inactivation [18], and wastewater treatment [19]. Their robustness and reusability render them particularly suitable for large‐scale or decentralized systems, where conventional enzymes or catalysts frequently prove inadequate. In the energy sector, nanozymes have been increasingly incorporated into electrochemical and photoelectrochemical platforms to catalyze essential reactions, including the oxygen reduction reaction (ORR) [20], hydrogen evolution reaction (HER) [21, 22], and carbon dioxide reduction reaction (CO_2_RR) [23, 24]. Furthermore, their multifunctional characteristics—encompassing redox activity, charge transport, and structural adaptability—have enabled the design of hybrid energy systems such as enzymatic biofuel cells and artificial photosynthetic assemblies.

Despite these advances, a number of critical issues remain unresolved. In particular, a fundamental mechanistic understanding of nanozyme catalysis at the atomic and molecular levels is still incomplete, which constrains the rational design of highly active and selective systems. Moreover, questions concerning catalytic specificity, long‐term stability, potential toxicity, and environmental persistence necessitate systematic investigation before broader technological implementation can be realized.

**SCHEME 1:**
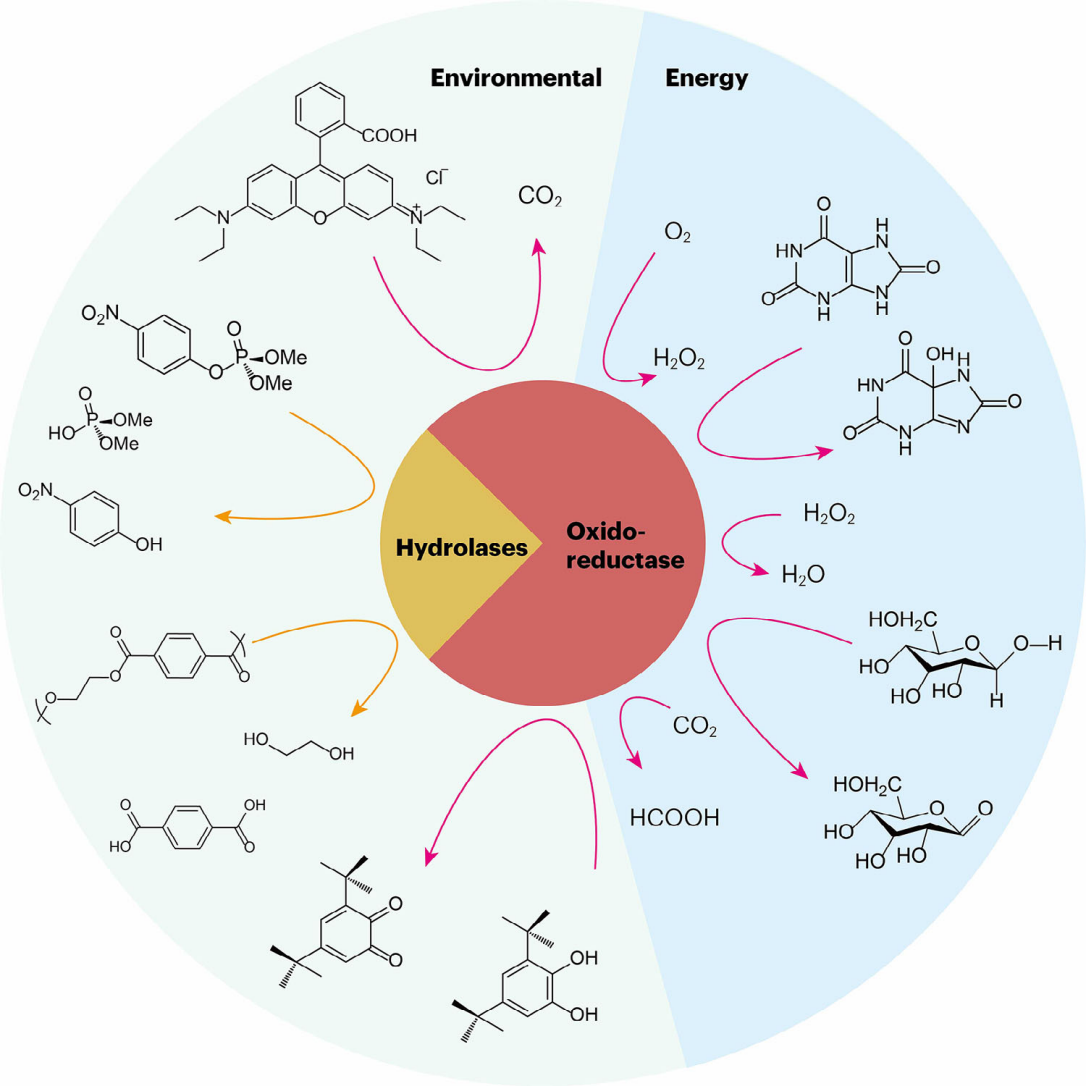
Nanozymes for energy and environmental applications by mimicking hydrolases and oxidoreductases.

This review provides a comprehensive account of recent progress in nanozyme research, with an emphasis on their roles in energy conversion and environmental sustainability. The discussion begins with a classification framework and current mechanistic insights, followed by an analysis of applications in environmental and energy‐related systems. The article concludes by identifying the principal challenges and outlining prospective research directions, with particular attention given to strategies that may expedite the transition of nanozyme‐based technologies toward practical sustainable applications (Scheme 1).

## 2 Energy

### 2.1 Nanozymes for H2O2 Production

**FIGURE 1:**
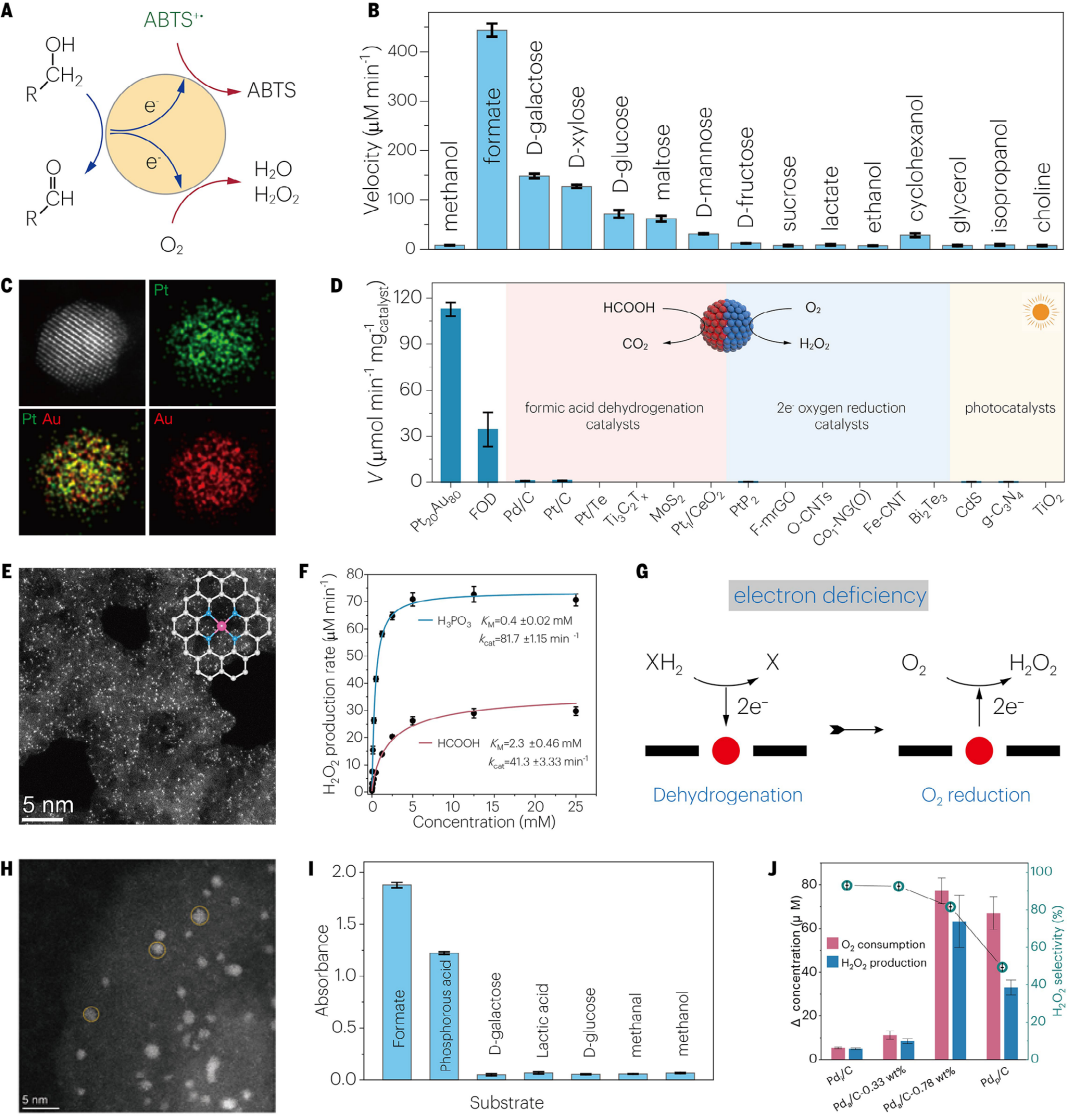
Nanozymes mimic flavoprotein enzymes for H_2_O_2_ production. A) Electron transfer mediated by a gold nanozyme. B, Au NP‐catalyzed reduction of ABTS**^+^** ^⚫^ in the presence of different substrates. A and B Reproduced with permission [25]. Copyright 2021, The Authors. C) AC‐HAADF‐STEM images of the Pt_20_Au_80_ alloy and corresponding elemental maps of Pt and Au. D) H_2_O_2_ production rates of different catalysts in 5 mM HCOOH and 5 mm HCOOK. C and D Reproduced with permission [26]. Copyright 2022, Wiley‐VCH GmbH. E) Atomic‐resolution HAADF‐STEM images of the Rh_1_/NC nanozyme. F) Initial H_2_O_2_ production rates (*V*_0_) for the Rh_1_/NC (10 µg mL^−1^)‐catalyzed oxidation of HCOOH and H_3_PO_3_. G) Schematic illustration of substrate dehydrogenation and the oxygen reduction reaction (ORR) catalyzed by single‐atom catalysts. E, F, and G Reproduced with permission [27]. Copyright 2022, The Authors. H) AC‐HAADF‐STEM images of Pd_a_/C. I) Absorbance at 652 nm after 15 min of reaction between Pd_a_/C‐0.78 wt.% (12.5 mg L^−1^) and various substrates (50 mm). J) O_2_ consumption and H_2_O_2_ production after 5 min of reaction in the presence of 30 mM HCOOH. H, I, and J Reproduced with permission [28] Copyright 2024, Chinese Chemical Society.

As an eco‐friendly oxidant, hydrogen peroxide (H_2_O_2_) plays a crucial role in industry, medicine, and the environment [29]. Compared with the anthraquinone process, electrocatalysis, and photocatalysis, organisms can catalyze the reduction of oxygen to generate hydrogen peroxide by flavoenzymes [30] in vivo under mild conditions. Flavoenzymes are a main type of oxidoreductase and include glucose oxidase (GOD), formate oxidase (FOD) and alcohol oxidase (AOD). Owing to the limitations of natural enzymes, some studies have reported the production of H_2_O_2_ by mimicking flavoenzymes. For example, Au nanoparticles (NPs) exhibit GOD‐like activity, and can catalyze the oxidation of glucose to produce H_2_O_2_. To explore the catalytic mechanism of the Au NPs, the 2,2′‐azino‐bis(3‐ethylbenzothiazoline‐6‐sulfonic acid) radical (ABTS^+⚫^), an electron acceptor that replaces O_2_, can be reduced in the presence of glucose (Figure 1A), which proves that the Au NPs have the same catalytic mechanism as GOD; that is, the Au NPs catalyze the oxidation of glucose by dehydrogenation instead of active oxygen radicals [25]. Furthermore, to explore the ability of the Au NPs to mimic other oxidases, some substances containing hydroxyl groups also underwent the same catalytic reaction, where formic acid (HCOOH) had the highest oxidation rate (Figure 1B), indicating that the Au NPs can also mimic FOD. On the basis of this conclusion and the advantages of HCOOH as a hydrogen storage material [31], a series of Pt_x_Au_100‐x_ alloy catalysts [26] with different gold‒platinum ratios were prepared to mimic FOD. In all the Pt_x_Au_100‐x_ samples, the yield of H_2_O_2_ shows a volcanic change with the increase of Pt content, among which Pt_20_Au_80_ exhibited the highest activity and the H_2_O_2_ productivity up to 7.2 mol g_cat_^−1 ^h^−1^. This is the result of the double action of electron donation by the PVP molecule and electron absorption by the Au NPs, which makes Pt_20_Au_80_ have a more reasonable *d*‐band center. High‐angle annular dark‐field scanning transmission electron microscopy (HAADF‐STEM) images and corresponding elemental mapps of the Pt_20_Au_80_ alloy show that the Pt and Au were uniformly distributed across the nanoparticles (Figure 1C). In addition, natural FOD and other related catalysts reported in the literature (formic acid dehydrogenation catalysts, 2‐electron oxygen reduction catalysts, and photocatalysts) all reacted under the same conditions as Pt_20_Au_80_ (Figure 1D). Pt_20_Au_80_ has three times the mass activity of natural FOD. Moreover, most of these catalysts do not produce H_2_O_2_ because they do not meet the necessary conditions for mimicking FOD at the same time: formic acid dehydrogenation and oxygen reduction. Notably, a major reason for the high yield and selectivity of Pt_20_Au_80_ is its low efficiency in H_2_O_2_ decomposition and high catalytic activity.

In recent years, single‐atom catalysts have been widely used in various fields because of their high activity and selectivity. A nitrogen‐doped carbon‐supported single‐atom rhodium catalyst (Rh_1_/NC) was reported to mimic flavin‐dependent oxidase, which was synthesized by simple pyrolysis with urea and polyethylene glycol as nitrogen and carbon, respectively [27]. The HAADF‐STEM image of Rh_1_/NC shows that Rh was evenly distributed across the carrier in the form of individual atoms (Figure 1E), and each Rh atom was coordinated with four N atoms. The kinetic curves of H_2_O_2_ produced at different concentrations with HCOOH and H_3_PO_3_ as substrates fit well with the Michaelis‒Menten equation, where Rh_1_/NC has a higher affinity for H_3_PO_3_ and better H_2_O_2_ productivity than does HCOOH (Figure 1F). DFT calculations revealed that the dehydrogenation of H_3_PO_3_ on Rh_1_/NC has a lower energy barrier, which further verifies the experimental results. Strikingly, commercial Pt/C, as a common 4‐electron ORR electrocatalyst, also produces a large amount of H_2_O_2_ via the enzymatic oxidation of H_3_PO_3_, which indicates that the enzymatic ORR is different from the electrocatalytic ORR. The reason for this is that the enzymatic ORR of Rh_1_/NC follows the ping‐pong mechanism, that is, Rh_1_/NC obtains two electrons and two hydrogens from the substrate and then combines with O_2_ for reduction to H_2_O_2_ (Figure 1G). Unlike the electrocatalytic ORR, which involves a continuous supply of electrons, the electron supply of the enzymatic ORR is limited by substrate dehydrogenation. On this basis, Xue et al. used Rh_1_/NC to mimic NADH oxidase and confirmed its ability to catalyze NADH oxidation and O_2_ reduction to generate H_2_O_2_ [32]. Moreover, Rh_1_/NC also exhibited obvious peroxidase‐like activity under acidic conditions. The multi‐enzyme activity of Rh_1_/NC can quickly consume intracellular NADH and induce metabolic disorders in cancer cells, which is expected to be useful in cancer treatment. This study has realized the generation and application of H_2_O_2_, but if Rh_1_/NC is applied in vivo, it is necessary to consider its targeting effect to improve its biological safety and therapeutic efficiency.

In addition, electrocatalysis studies have revealed that Rh single atoms have a high catalytic ability for HCOOH oxidation [33]. For comparison, palladium (Pd) single atoms have almost no activity. However, Pd single atoms are highly selective for the synthesis of H_2_O_2_ [34] and are less expensive than Rh is. Moreover, Pd is the main catalyst for the production of H_2_O_2_ in industry. On this basis, Pd single‐atom assembly (Pd_a_) has been explored as a new FOD mimic [28]. Figure 1H shows the distribution of Pd atoms in the assembly, which is formed by the irregular arrangement of several single atoms. Furthermore, the colorimetric absorbance of Pd_a_, which catalyzes the oxidation of different substrates to produce H_2_O_2,_ shows that Pd_a_ has the highest activity for HCOOH (Figure 1I), which is different from the high activity of Rh_1_/NC for H_3_PO_3_. The H_2_O_2_ selectivity of Pd_a_ and Pd nanoparticles was also tested, in which Pd_a_ had higher selectivity than did the nanoparticles (Figure 1J). Therefore, the high FOD‐like activity of Pd_a_ is due to its high HCOOH oxidation ability and H_2_O_2_ selectivity. Similarly, inspired by metalloenzymes, bismuth nanoclusters (Bi NCs) confined within mesoporous carbon channels, which outperform conventional bismuth nanoparticles (Bi NPs) for selective 2‐electron O_2_ reduction to H_2_O_2_ under neutral conditions, have been reported [35]. Compared with larger Bi NPs that favor strong ^*^OOH binding and the 4‐electron ORR pathway, ultrasmall Bi NCs exhibit a distinct electronic structure that optimizes ^*^OOH adsorption and facilitates its rapid desorption as H_2_O_2_, thereby suppressing further reduction. In addition to the intrinsic advantages of cluster‐sized active sites, the mesoporous microenvironment plays a decisive role by simultaneously enhancing O_2_ accessibility and creating a locally alkaline reaction zone through OH^–^ accumulation. The synergistic coupling between Bi NCs and mesoporous channels closely resembles metalloenzyme architectures, where metal clusters and surrounding microenvironments cooperatively govern catalytic efficiency, offering a general design principle for advanced electrocatalysts.

Therefore, the nanozyme for synthesizing H_2_O_2_ must meet two conditions: one is that it can oxidize the substrate, and the other is that it has certain a 2‐electron O_2_ reduction selectivity. The Bi NCs mentioned above do not belong to this type of nanozyme, because they only carry out the second half of the reaction and do not oxidize the substrate, even though their design is inspired by the enzyme.

### 2.2 Nanozymes for Electrocatalysis

The exceptional catalytic efficiency of natural enzymes, together with rapid progress in nanozyme research, has motivated the development of enzyme‐inspired nanostructures for electrocatalysis. By integrating structural motifs of enzymatic active centers into synthetic frameworks, researchers have constructed nanozymes capable of driving key electrocatalytic transformations with markedly enhanced activity and selectivity. Studies have shown that nanozymes can effectively catalyze key reactions in electrochemical processes, such as carbon dioxide reduction (CO_2_RR), nitrate reduction (NO_3_RR), hydrogen evolution (HER), and oxygen reduction (ORR).

#### 2.2.1 Nitrate reduction

**FIGURE 2:**
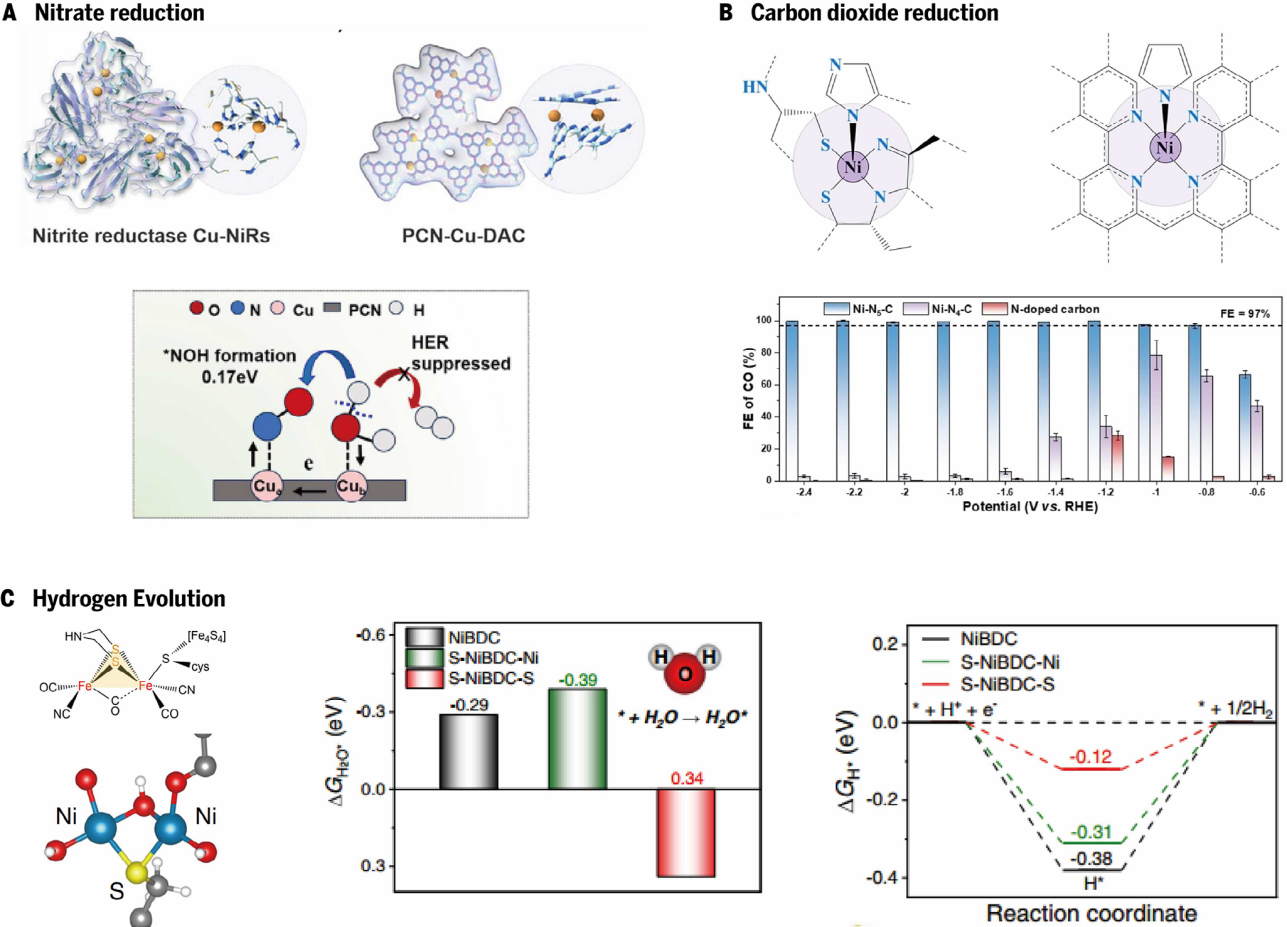
Nanozymes for electrocatalysis. A) Illustration of dual Cu atomic sites in natural nitrite reductases (Cu‐NiRs) and the PCN‐Cu‐DAC nanozyme and nitrate reduction mechanism of PCN‐Cu‐DAC. Reproduced with permission [36]. Copyright 2025, Wiley‐VCH GmbH. B) Comparison of Ni–N active sites in natural Ni‐SOD and a single‐atom nanozyme, along with the Faradaic efficiencies for CO production. Reproduced with permission [24]. Copyright 2022, Wiley‐VCH GmbH. C) Active site structure of [FeFe]‐hydrogenase, schematic of the “Ni_2_–S_1_” active region in S‐NiBDC, and Free‐energy diagrams for water adsorption and H adsorption of NiBDC and S‐NiBDC. Reproduced with permission [22]. Copyright 2022, The Authors.

Ammonia serves as a fundamental feedstock for the fertilizer industry and numerous basic organic chemicals; however, conventional synthesis routes are highly energy intensive and are accompanied by substantial CO_2_ emissions. In this context, the NO_3_RR has emerged as a sustainable pathway for ammonia synthesis, offering a green alternative to energy‐intensive Haber–Bosch processes [37]. The NO_3_RR is a multielectron and multiproton transfer process [38]. At present, the imbalance between protonation and the HER has been solved by the cooperation of multiple metal sites, but there are still issues of low yield and poor stability. To address these challenges, an enzyme‐inspired dual‐atom copper anchored on a polymeric carbon nitride (PCN‐Cu‐DAC) catalyst was developed (Figure 2A) [36]. This design mimics nitrite reductase, where adjacent Cu sites synergistically promote nitrate adsorption, proton transfer, and ^*^NO protonation, thereby lowering the energy barrier for ^*^NO → ^*^NOH. Specifically, upon nitrate adsorption, the dual Cu site configuration provides a stronger interaction with NO_3_^–^ than the single Cu site dose, facilitating its stepwise deoxygenation to ^*^NO. The adsorption of ^*^NO induces an asymmetric electronic redistribution within the Cu pair, leading to electron accumulation on Cu_a_ and electron depletion on the neighboring Cu_b_. Such dynamic polarization results in a functional division of labor, where the electron‐rich Cu_a_ preferentially stabilizes ^*^NO, whereas the electron‐deficient Cu_b_ promotes water dissociation and ^*^H generation. Owing to the short interatomic distance between the Cu centers, the generated *H can be rapidly transferred to the adjacent ^*^NO species, significantly lowering the energy barrier for the potential‐determining ^*^NO → ^*^NOH protonation step. This efficient proton delivery suppresses ^*^NO accumulation and minimizes competitive HER, thereby enabling continuous hydrogenation and deoxygenation of nitrogen intermediates toward selective NH_3_ formation. The experimental results also revealed that dual‐site cooperation optimizes the electron distribution, accelerates ^*^H transfer from water dissociation, and suppresses competing hydrogen evolution. Compared with Cu single‐atom and Cu nanoclusters, PCN‐Cu‐DAC has the lowest onset potential and superior activity, achieving nearly 98% FE and gram‐scale ammonia yields under industrially relevant conditions. The dual Cu site plays two roles: the adsorption of NO_3_^–^ and the generation of ^*^H, which is the key to improving efficiency. By extension, these are not just bimetallic sites, but also bifunctional sites, therefore, the design idea of the catalyst is to realize these two functions, and the current mainstream is to accelerate the generation and transfer of ^*^H, such as the intrinsic Ni^0^–Ni^δ+^ pairs of NiNNi_3_/Cu and pyridine axial coordination of ACCs‐Fe.

#### 2.2.2 Carbon Dioxide Reduction

Converting CO_2_ into valuable chemicals or fuels is a promising strategy for effectively utilizing CO_2_ to reduce carbon emissions and achieve carbon neutrality [39]. Recently, the electrocatalytic reduction of CO_2_ driven by renewable electricity has emerged as a highly attractive strategy for sustainable carbon conversion [40]. To improve the long‐term stability and reaction rate and reduce catalyst costs, Huang et al. reported a single‐atom nanozyme with square‐pyramidal Ni–N_5_ sites for the eCO_2_RR (Figure 2B) [24].The unique coordination geometry and electronic microenvironment of the metal centers in natural Ni‐SOD inspired the design of Ni–N_5_–C. In a CO_2_‐saturated 0.5 m KHCO_3_ solution, the Faraday efficiency (FE) of CO_2_‐to‐CO conversion catalyzed by Ni–N_5_–C is as high as 99.6% at −2.4 V vs. RHE. For comparison, Ni–N_4_–C and N‐doped carbon were synthesized for testing. The linear sweep voltammetry curves (LSV) and FE results demonstrated that Ni─N_5_─C has a higher performance than the other catalysts do and exceeds the requirements at the industrial level. Density functional theory (DFT) calculations revealed an increase in the d_Z2_ orbital energy level in Ni─N_5_─C compared with that in Ni─N_4_─C, which promoted CO_2_ activation and facilitated CO desorption, thereby achieving superior selectivity, activity, and durability over traditional catalysts. This square‐pyramidal structure of the Ni─N_5_ is similar to that of ACCs‐Fe which also has axial coordination for the NO_3_RR, and both catalysts are used to avoid the HER. The HER is a common problem faced by the CO_2_RR and NO_3_RR, so the catalyst design of these two reactions has the significance of learning from each other, and it is not difficult to find that some similar catalysts have been reported.

#### 2.2.3 Hydrogen Evolution

Hydrogen is widely regarded as a clean and sustainable alternative to fossil fuels, and its strategic importance in future energy systems continues to grow [41]. For reasons of cost‐effectiveness and operational safety, large‐scale industrial hydrogen production via water electrolysis predominantly employs alkaline electrolytes. Inspired by the high efficiency “Fe_2_S_1_” active sites in [FeFe]‐hydrogenase, Cheng et al. developed a sulfur‐modulated Ni‐benzenedicarboxylic acid‐based MOF (S‐NiBDC) catalyst to overcome the sluggish kinetics of hydrogen evolution in alkaline media [22]. In S‐NiBDC, S atoms bridge two Ni centers to construct a triangular “Ni_2_─S_1_” motif resembling the enzymatic Fe_2_─S_1_ site (Figure 2C), breaking the symmetric metal coordination of conventional MOFs and improving the catalytic performance. Mechanistic studies combining spectroscopy and DFT calculations confirmed that the Ni sites in S‐NiBDC accelerate the adsorption and activation of water, and the ΔG_H*_ of the S sites in S‐NiBDC is closer to the optimal value which enables S to trap hydrogen atoms, thereby facilitating the Volmer–Heyrovsky pathway. Furthermore, S modulation improved the strength of the Ni─O bond, resulting in outstanding structural stability (over 150 h at 1.0 A cm^−2^) at industrial‐level current densities. These results reveal that the synergistic Ni─S configuration enhances electron transfer, stabilizes the framework, and lowers the energy barrier for hydrogen adsorption and evolution. Compared with pristine NiBDC and commercial Pt/C, S‐NiBDC has a markedly lower overpotential (310 mV at 1.0 A cm^−2^) and a small Tafel slope of 75 mV dec^−1^. Compared with the NO_3_RR catalyst, this Ni_2_─S_1_ site plays the same role as Cu_a_ in PCN─Cu─DAC and Ni^0^–Ni^δ+^ pairs of NiNNi_3_/Cu, that is, it accelerates hydrolysis and dissociation to generate ^*^H [42, 43]. Therefore, replacing one nickel in Ni_2_‐S_1_ with copper to form a Cu─S─Ni structure may be a better NO_3_RR catalyst.

These work demonstrate how enzyme‐inspired structural engineering provides a universal strategy to construct highly active and durable electrocatalysts beyond noble metals.

### 2.3 Nanozymes for Biofuel Cells

**FIGURE 3:**
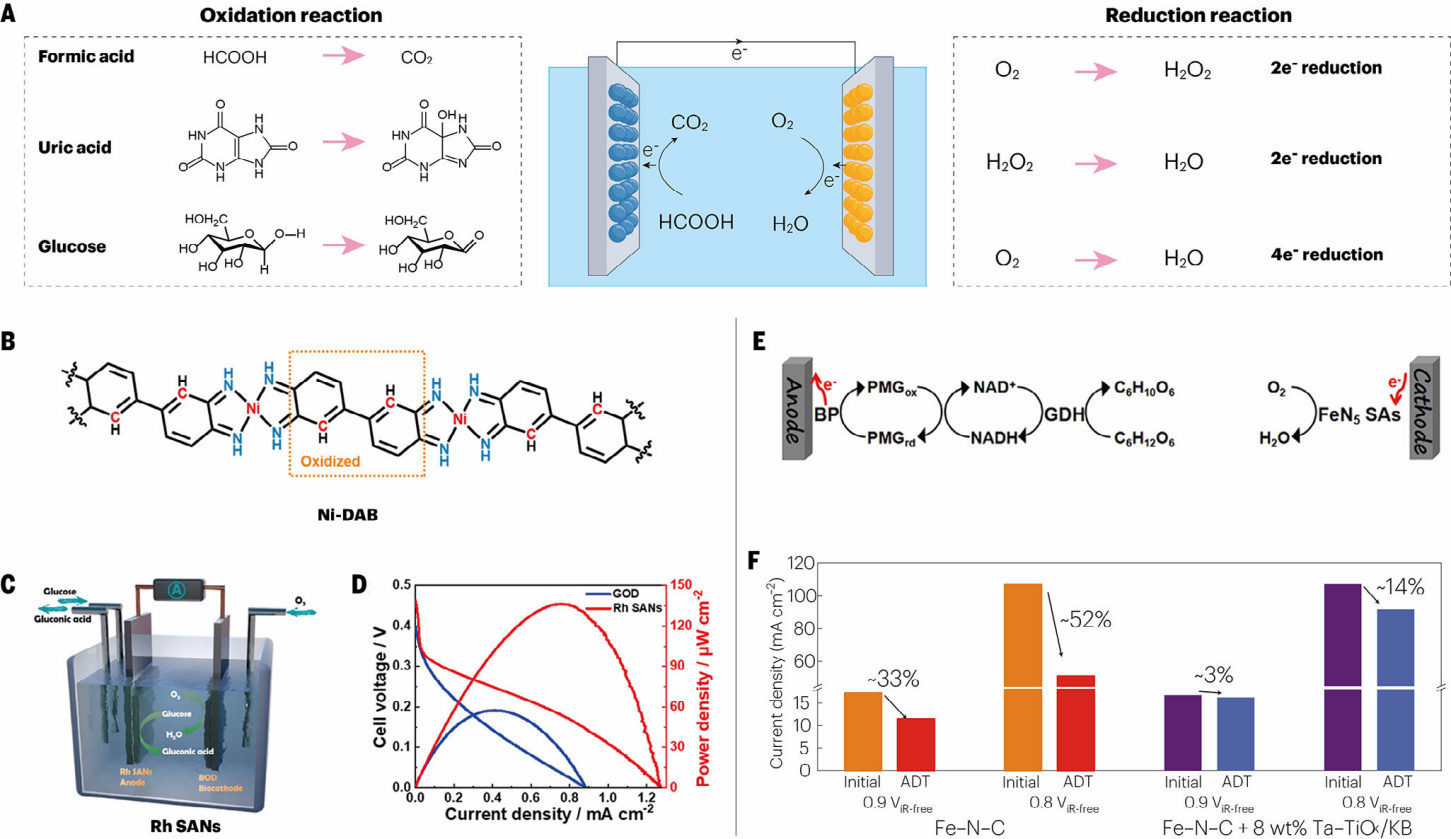
Nanozymes for biofuel cells. A) Schematic illustration of the cathodic and anodic reactions in a biofuel cell. B) Coordination structure of the Ni‐DAB complex. Reproduced with permission [44]. Copyright 2024, The Authors. C) Schematic representation of the Rh single‐atom nanosheets (Rh SANs)‐based glucose/O_2_ enzymatic biofuel cell setup. D) Power density versus current density curve measured in O_2_‐saturated 0.1 m PBS (pH 7.0) containing 100 mm glucose. C and D Reproduced with permission [45]. Copyright 2023, American Chemical Society. E) Schematic diagram of a glucose/O_2_ EBFC employing FeN_5_ single atoms (SAs) as the cathode catalyst. Reproduced with permission [46]. Copyright 2021, American Chemical Society F) Comparison of current density decay for cells with and without Ta─TiO_x_/KB after the ADT. Reproduced with permission [47]. Copyright 2022, The Authors.

A biofuel cell (BFC) is an electrochemical device that directly converts the chemical energy of biomass into electrical output, distinguished by the utilization of biological catalysts (such as enzymes or microorganisms) to mediate electrode reactions. As shown in Figure 3A, their operation relies on two half‐reactions occurring at the anode and cathode: at the anode, a fuel (such as formic acid, uric acid, or glucose) is oxidized by biocatalysts, releasing electrons and protons; at the cathode, an oxidant (typically O_2_) accepts these electrons and protons, which are subsequently reduced to H_2_O_2_ or H_2_O. For example, in HCOOH BFC [48], electrons are transferred to the cathode surface either via mediators or through direct electron transfer pathways. The cathode reaction commonly employs enzymes (e.g., laccase, bilirubin oxidase) or abiotic catalysts to facilitate the four‐electron reduction of O_2_ to H_2_O. The distinctive advantages of BFCs are their mild operating conditions (ambient temperature and pressure, near‐neutral pH), excellent biocompatibility, and ability to utilize widely available biomass resources. These features present significant application potential in fields such as implantable medical devices, environmental monitoring, and integrated waste‐to‐energy systems.

#### 2.3.1 Nanozymes for Fuel Cell Anodes

Nanozymes have recently emerged as promising alternatives to natural biocatalysts for application in biofuel cells (BFCs). Wang et al. developed a metal–ligand dual‐site single‐atom nanozyme (Ni‐DAB), which mimics the dual‐site catalytic mechanism of urate oxidase (UOX) [44]. The Ni‐DAB catalyst was synthesized by coordinating Ni centers with 3,3′‐diaminobenzidine ligands, forming an atomically dispersed coordination polymer (Figure 3B). The Ni metal center selectively binds uric acid (UA), while the β‐carbon atoms of the ligand bind molecular O_2_, thereby mimicking the isolated binding pockets of UOX, which was confirmed by spectroscopic analyses, isotope labeling, and DFT calculations. This dual‐site design endows Ni‐DAB with remarkable substrate selectivity, enabling efficient UA oxidation while minimizing activity toward competing substrates such as glucose, dopamine, and phenolics. Compared with conventional Pt/C catalysts, Ni‐DAB displayed superior catalytic efficiency, stability over a broad pH and temperature range, and threefold higher specific activity. Furthermore, the incorporation of carbon black (Ni‐DAB/C) increased the accessible surface area and further improved the specificity by suppressing side reactions. The molecular selectivity toward UA arises from its favorable electronic and coordination compatibility with the isolated Ni─N_4_ site. Deprotonated urate coordinates to the Ni center via the C8 carbonyl oxygen, enabling efficient electron donation and a moderate, reversible binding that initiates interfacial electron transfer. In contrast, glucose lacks conjugated carbonyl groups and remains electronically saturated, rendering its hydroxyl functionalities ineffective for coordination or electron transfer. Consequently, glucose fails to activate the coupled O_2_ reduction pathway, leading to the intrinsic selectivity of the catalyst for uric acid. This catalyst once again proves the importance of double‐site division of labor, which is a new strategy to improve the catalytic efficiency of nanozymes. NanozymeS with a double‐site have been introduced many times in different fields, which shows that they have high research value. The practical utility of Ni‐DAB was validated in a biofuel cell (BFC) using UA from human urine as fuel, achieving a maximum power density of 65 µW cm^−2^ and an open‐circuit potential of 0.40 V, outperforming Pt‐based counterparts. Importantly, the device successfully powered a temperature and humidity sensor, demonstrating stable performance in real urine samples. In addition, glucose/O_2_ enzymatic biofuel cells (EBFCs) have attracted widespread attention because of their advantages in terms of environmental protection. Zhao et al. introduced rhodium single‐atom nanozymes (Rh SANs), synthesized via a one‐step pyrolysis route, as a novel class of GOD mimics [45]. Mechanistic investigations demonstrated that Rh SANs mimic the dehydrogenation oxidation pathway of natural GOD, mediating the release of two electrons and two protons during glucose oxidation. Importantly, unlike other noble/transition metal catalysts, Rh SANs also catalyze the direct two‐electron electrooxidation of glucose to gluconic acid at neutral pH. The electrocatalytic performance of Rh SANs was validated by integration into a glucose/O_2_ EBFC with a bilirubin oxidase cathode (Figure 3C). The device delivered a maximum power density of 135 µW cm^−2^, which was 2.35 times greater than that of a GOD‐based BFC (Figure 3D). Notably, the EBFC can be fuelled directly by commercially available beverages such as coffee and soft drinks, illustrating the feasibility of low‐cost and accessible biofuels. Furthermore, device miniaturization was demonstrated through fabrication on flexible PET‐ITO substrates, producing a portable prototype with an output of 43.4 µW cm^−2^ at 0.20 V, thereby underscoring the potential of Rh SANs‐based EBFCs in powering portable, wearable, and implantable electronics.

#### 2.3.2 Nanozymes for Fuel Cell Cathodes

##### 2.3.2.1 Oxidase Activity and Oxygen Reduction

All of the above studies focused on the anode reaction, and improving the cathode oxygen reduction activity is also the key to improving the fuel cell power density. Oxidase is a type of oxidoreductase that needs O_2_ to catalyze the oxidation of organic substrates, in which O_2_ undergoes a reduction reaction to produce water or H_2_O_2_. In recent years, oxidase‐like materials, as substitutes for expensive natural oxidases, have been applied in many fields and have broad development prospects [49]. Lu et al. proposed a simple and novel method to evaluate the activity of oxidase‐like materials, which can quickly screen materials with potential oxidase‐like properties [50]. They reported that there is a close correlation between oxidase‐like activity and ORR electrocatalysis, both of which rely on similar catalytic origins. To verify this hypothesis, Cu and Fe atoms were coanchored on nitrogen‐doped carbon supports (CF‐HNCS) to create well‐defined active sites inspired by natural cytochrome c oxidases (CcO). Structural characterization confirmed the existence of Cu─Fe dual sites, mimicking the heterobinuclear centers of natural enzymes. The chromogenic reactions and LSV curves of the CF‐HNCS and reference samples show that CF‐HNCS not only exhibited strong oxidase‐like behavior but also superior ORR activity, demonstrating a linear relationship between the oxidase‐like catalytic efficiency and ORR performance and enabling the use of electrochemical measurements as reliable descriptors of enzymatic activity. Density functional theory calculations revealed that Cu facilitated O_2_ adsorption and activation, whereas Fe promoted water release, together lowering the overpotential. However, it is worth noting that this conclusion is suitable only for oxidase mimics in which O_2_ is activated first and then oxidizes the substrate and is not suitable for flavoenzyme mimics in which O_2_ is used only as an electron acceptor (such as the nanozymes used for H_2_O_2_ generation as mentioned above). Likewise, Zhang et al. also designed CcO‐like single‐atom nanozymes (FeN_5_ SAs) for the oxygen reduction reaction (ORR) in EBFCs [46]. The FeN_5_ SAs were integrated as cathodes in glucose/O_2_ EBFCs paired with a glucose dehydrogenase (GDH) bio‐anode modified with a polymethylene green mediator (Figure 3E), achieving an open‐circuit potential of 0.40 ± 0.01 V and a maximum power density of 149.2 ± 4.0 µW cm^−2^ under O_2_‐saturated conditions—approximately 3.8 times higher than those of Pt/C‐based EBFCs. Importantly, the EBFC retained 91% of its initial power output after 20^,^000 s of operation, underscoring the robustness of the system. This excellent activity stems from the fact that FeN_5_ SAs mimic the axial coordination site of natural CcO, which offers a rational framework for developing next‐generation nanozymes through atomic‐level biomimetic engineering.

##### 2.3.2.2 Nanozymes for Improving Electrocatalytic Stability

The ORR is a key process for fuel cells, in which the most commonly used catalysts are platinum (Pt)‐based catalysts with high activity and stability [51]. However, the high cost of Pt makes platinum‐group‐metal‐free (PGM‐free) catalysts such as Fe─N─C widely studied as substitutes for platinum‐group materials [52, 53]. While these catalysts show promising activity and low cost, their rapid degradation in acidic environments hinders their practical application. The primary causes of instability include demetallation, carbon corrosion, and attack from reactive oxygen species (ROS), such as hydroxyl (⚫OH) and hydroperoxyl (HO_2_⚫) radicals, which originate from incomplete oxygen reduction and H_2_O_2_ intermediates. Traditional stabilization strategies have focused on increasing graphitization of the carbon matrix or minimizing metallic particle dissolution. However, these methods either reduce the catalytic activity or only partially suppress degradation.

To overcome this limitation, Xie et al. reported an active defense approach involving the incorporation of Ta–TiO_x_ nanoparticles as radical scavengers [47]. These nanoparticles are approximately 5 nm in size and are uniformly distributed on a carbon substrate. Experimental evidence from fluorescence spectroscopy, electron paramagnetic resonance (EPR) measurements, and rotating ring disk electrode (RRDE) tests confirmed that Ta–TiO_x_ significantly reduces the radical concentration and decreases the H_2_O_2_ yield. In particular, Ta–TiO_x_ nanoparticles with a rutile TaO_2_ phase have superior scavenging performance, decreasing the H_2_O_2_ yield by nearly 51% at 0.7 V. During fuel cell operation, the cell current density (Figure 3F) of the Fe─N─C catalyst with or without Ta–TiO_x_ scavengers before and after the accelerated durability test (ADT) revealed that the scavenger‐containing system exhibited minimal performance loss, with a 3% current density decay at 0.9 V_iR‐free_, whereas the corresponding value was 33% for the control. Density functional theory (DFT) calculations further revealed that the TaO_2_–OH surface provides strong adsorption energies for ROS, facilitating their decomposition through OH^*^‐ and O^*^‐assisted pathways. Typically, in terms of free radical scavenging, nanozymes with SOD‐like and CAT‐like activities have been widely studied and applied because of their ability to scavenge free radicals [54, 55], which may achieve the same effect as Ta–TiO_x_. Qiu et al. combined FeNC with cerium oxide (CeO_2_) nanozymes and improved the ORR durability of FeNC, in which CeO_2_ was employed to actively scavenge harmful ROS such as H_2_O_2_, O_2_^⚫−^, and ⚫OH [20]. Control experiments revealed that introducing CeO_2_ had no influence on the catalytic activity. In addition, after 10,000 accelerated cycles, the E_1/2_ of FeNC/CeO_2_ decayed by only 5 mV, whereas it was 18 mV for FeNC and 14 mV for Pt/C. In situ electron paramagnetic resonance (EPR) and scanning electrochemical microscopy (SECM) confirmed that CeO_2_ effectively eliminated ROS in situ, protecting Fe active sites from demetallization and carbon corrosion. When applied in zinc–air batteries, the FeNC/CeO_2_ catalyst delivered a peak power density of 66.15 mW cm^−2^ and stable cycling over 140 h, significantly outperforming both FeNC and Pt/C. In summary, the use of Ta–TiOx nanoparticles and CeO_2_ transforms catalyst protection from passive stabilization to an active defense strategy, offering a generalizable method to increase the stability of PGM‐free ORR catalysts.

### 2.4 Nanozymes for Lignin Degradation

**FIGURE 4:**
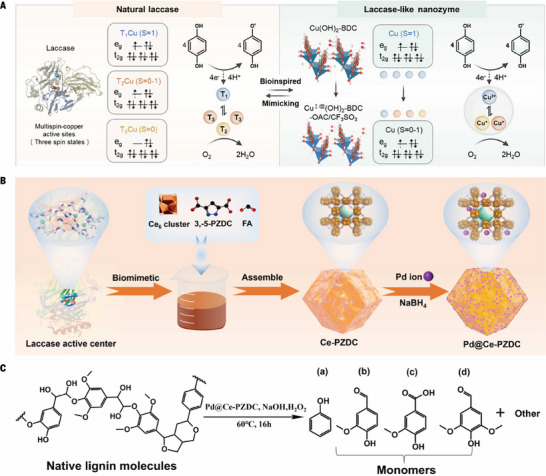
Nanozymes mimicking laccases for biomass degradation. A) Schematic illustration of the multispin‐state characteristics of natural laccase multicopper centers and a 2D copper‐based MOF COHBLO with optimal laccase‐like activity. Reproduced with permission [56]. Copyright 2025, Wiley‐VCH GmbH. B) Synthesis and morphological characterization of Ce‐PZDC and Pd@Ce‐PZDC nanozymes. C) Schematic illustration of the lignin depolymerization pathway catalyzed by Pd@Ce‐PZDC. B and C Reproduced with permission [57]. Copyright 2025, American Chemical Society.

Lignin degradation into high‐value aromatic compounds has been extensively investigated as a strategy to reduce reliance on fossil resources. Conventional approaches, such as pyrolysis and hydrogenolysis, generally require harsh reaction conditions [58, 59]. In natural systems, white‐rot fungi secrete laccase, lignin peroxidase, and manganese peroxidase, which act synergistically to degrade lignin through the cleavage of carbon–carbon and ether linkages [60]. The enzymatic degradation of lignin offers significant advantages, such as mild reaction conditions and high efficiency, but challenges, such as complex purification, enzyme instability and deactivation, restrict its degradation efficiency, remain. To solve this problem, the design of nanozymes based on natural systems has been widely studied. At present, most studies have focused on simulating single enzyme activity, for example, Yu et al. reported a copper‐based nanozyme system (Cu(OH)_2_BDC‐LA‐OAc (COHBLO)) with high selectivity for lignin depolymerization through spin state regulation, and its design idea stems from the multispin synergistic mechanism of multi‐copper sites in natural laccases (Figure 4A) [56]. By combining redox modulation and ligand‐field engineering within an ultrathin 2D MOF architecture, the copper centers are driven into distinct yet coexisting spin configurations. This structural precision enables a systematic correlation between the copper spin state and catalytic performance, ultimately revealing a volcano‐type dependence of laccase‐like activity on the average spin magnetic moment. Mechanistically, an intermediate copper spin state optimizes the balance between electron localization and delocalization at the active centers. Density functional theory calculations demonstrate that this spin regime upshifts the Cu d‐band center and strengthens Cu─O_2_ orbital hybridization, thereby enhancing oxygen adsorption and interfacial charge transfer. Excessively high spin states suffer from over‐localized electrons that weaken cooperative oxygen activation, whereas low‐ or zero‐spin configurations suppress O_2_ activation due to insufficient unpaired electrons. Consequently, only the multispin coexistence state maximizes electron flow across adjacent Cu sites, closely mimicking the cooperative T_1_/T_2_/T_3_ copper centers of native laccases. Enhanced oxygen activation triggers a controlled reactive oxygen species cascade, which selectively oxidizes lignin β‐O‐4 ether linkages while minimizing uncontrolled radical recombination. As a result, lignin was directionally depolymerized into low‐molecular‐weight fragments enriched in phenolic hydroxyl groups rather than randomly cleaved or over‐oxidized products within 120 h which is convenient for the subsequent preparation of excellent lignin adhesives.

Unlike the synergetic enzyme system for lignin degradation in nature, single‐site nanozymes are not conducive to the degradation of complex lignin macromolecules. Jiang et al. developed a dual‐site biomimetic catalyst, “Pd@Ce‐PZDC”, with laccase‐like and peroxidase‐like properties by combining Ce‐MOFs with Pd NPs (Figure 4B) [57]. and achieved the synergistic degradation of lignin. Among them, 3,5‐pyrazole dicarboxylic acid (3,5‐PZDC) was selected as the ligand to better simulate the histidine residue of the laccase active site, and formic acid (FA) created oxygen vacancy defects, thereby enhancing the laccase‐like activity of Ce‐PZDC. Michaelis–Menten kinetic analysis revealed that Pd@Ce‐PZDC has a lower *K*_m_ and *V*_max_ than Ce‐PZDC and laccase. The dual‐site interaction, supported by density functional theory (DFT), means that Pd not only contributes to peroxidase‐like reactivity but also activates Ce(IV) to behave similarly to Ce(III), thereby expanding the density of active sites and increasing overall efficiency. This cooperative mechanism enables the selective cleavage of β‐O‐4 and β−β linkages in lignin under mild aqueous conditions. After 16 h of reaction at 60°C, the major products identified included aromatic aldehydes such as syringaldehyde and vanillin and acids such as vanillic acid (Figure 4C). The total yield of these aromatic monomers is approximately 13.0%, which is significantly greater than the yields reported with other methods. This dual‐site approach offers a promising pathway for lignin valorization, presenting a more efficient and sustainable solution than traditional methods do. This highlights the potential of bioinspired, multifunctional catalysts for enhancing lignin depolymerization, with broad applications in biofuel and bioproduct production. It is not difficult to find that H_2_O_2_ needs to be added in this catalytic degradation process. But it will be a big breakthrough if H_2_O_2_ can be “self‐produced and self‐sold”. Although the flavoenzymes‐like mentioned above can produce H_2_O_2_, the oxidation of specific substrates is necessary, and lignin does not provide this condition. Perhaps a highly active photocatalyst (such as COF) can generate H_2_O_2_ without a specific substrate, further increasing the degradation efficiency.

Compared with the degradation time of COHBLO, the degradation time of Pd@Ce‐PZDC is shorter (the influence of temperature is not considered here). This clearly shows that the effect of multiple enzymes is better than that of a single enzyme, because laccase is mild and can easily to generate dimers, whereas peroxidase can greatly improve the reaction rate. Nanozymes with multi‐enzyme activity are mostly aggregates of nanozymes with different activities. Notably, some catalysts exhibit different enzyme‐like activities when the reaction conditions are adjusted which is also the biggest problem faced by nanozymes: poor specificity.

## 3 Environment

### 3.1 Nanozymes for Plastic Degradation

**FIGURE 5:**
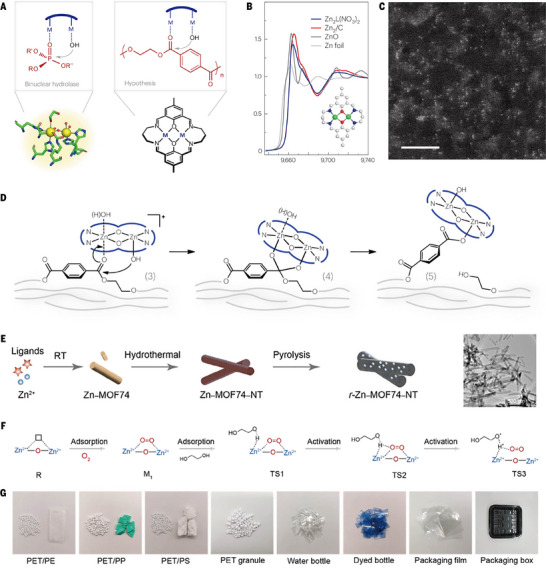
Nanozymes for plastic degradation. A) Schematic representations of the active sites and reaction mechanisms proposed for the binuclear hydrolase OpdA and the hypothesized PET‐degrading binuclear catalyst. B) Normalized XANES spectra of Zn_2_L(NO_3_)_2_, Zn_2_/C and references. C) The aberration‐corrected HAADF‐STEM image of Zn_2_/C. D) The mechanism of PET hydrolysis over the binuclear catalyst. A, B, C, and D Reproduced with permission [61]. Copyright 2023, The Authors. E) Synthesis and structural characterization of r‐Zn–MOF74–NT. F) Schematic diagram of the change in the atomic structure of O_2_ and EG on r‐Zn–MOF74–NT. G) Photographs of polyester waste. E, F, and G Reproduced with permission [62]. Copyright 2025, Wiley‐VCH GmbH.

The excessive use of plastic products has long been recognized as a serious threat to ecosystems and human health. Conventional waste management practices, such as landfilling and incineration, merely transfer the problem while imposing additional environmental burdens, thereby underscoring the urgent need for efficient and sustainable recycling strategies [63]. Mechanical recycling provides a straightforward route for plastic waste treatment, but its applicability is limited by issues such as contamination and product downgrading. In pursuit of a sustainable circular economy, chemical depolymerization—which converts polymers back into reusable monomers—has emerged as a more promising strategy. However, conventional chemical recycling typically requires harsh conditions such as high temperatures, strong acids or bases, and high pressure, all of which increase energy costs and generate secondary products [64]. Zhang et al. designed a biomimetic binuclear zinc catalyst (Zn_2_L(NO_3_)_2_) inspired by the active sites of organophosphate‐degrading enzyme (OpdA) [61]. OpdA employs two closely positioned metal centers to accelerate hydrolysis. These metal ions coordinate both the nucleophile (usually hydroxide) and the phosphate, bringing them into proximity and thereby increasing the local concentration of reactive species (Figure 5A), which is similar to the proximity effect of PETases degrading PET. XAS (Figure 5B) and HAADF‐STEM (Figure 5C) revealed that two zinc atoms of Zn_2_L(NO_3_)_2_ are linked by bridging O atoms to form a diatomic catalyst with no Zn‒Zn bonds, which accurately mimics the metal active center structure of OpdA. The catalyst exhibited significant activity at pH 8 and 40°C, achieving complete PET depolymerization within weeks, whereas the common zinc acetate and zinc oxide were inactive under the same conditions. Compared with commercial enzymes such as Humicola insolens cutinase, which often plateau at partial conversions due to product inhibition or denaturation, the binuclear zinc catalyst maintained robust activity even with high‐crystallinity PET. Besides, it also has activity in seawater, highlighting its potential for environmental remediation. At optimized conditions (pH 13, 90°C), the catalyst achieved specific activities exceeding 577 g_PET_ h^−1^ g_cat_^−1^, showing higher activity under milder conditions than other processes. The exploration of activity revealed that there was an induction period in the catalytic process, during which Zn_2_L(NO_3_)_2_ gradually transformed into the active hydroxyl‐bridged species (Zn_2_L(OH)_2_, as shown in Figure 5D (3)). Once this transformation is complete, the catalyst operates through a cycle of substrate binding, stabilization of a six‐membered transition state, bond cleavage, and regeneration of the active center. Moreover, closed‐loop recycling of post‐consumer PET into bottle‐grade PET was successfully demonstrated, confirming the feasibility of high‐value recycling.

In addition, the catalytic glycolysis of polyethylene terephthalate (PET) has received increasing attention due to its potential to generate bis(hydroxyethyl) terephthalate (BHET), which is a direct monomer for synthesizing PET and can realize closed‐loop regeneration of PET. Cao et al. developed a reconstructed Zn–MOF74 nanotube catalyst (r‐Zn–MOF74–NT) via dissolution–recrystallization and pyrolysis‐reconstruction methods (Figure 5E) [62]. TEM images of each synthesis stage revealed structural changes, and r‐Zn–MOF74–NT was composed of uniform zinc oxide nanoparticles. The resulting material displayed abundant zinc defect sites that facilitated oxygen and ethylene glycol activation, leading to the formation of nucleophilic intermediates (Figure 5F). These species effectively cleave polyester C─O bonds through nucleophilic attack, enabling efficient BHET production under mild conditions. The experimental results show that r‐Zn–MOF74–NT (5 wt.%) achieved 100% PET conversion with a BHET yield of 92.4%, outperforming traditional catalysts such as commercial ZnO. Theoretical calculations show that the energy barrier (0.42 eV) for activation by EG and O on a surface rich in zinc defects is much lower than that on a perfect ZnO surface (1.28 eV). This finding directly proves that the existence of defects greatly promotes the activation process. In addition, through the identification of glycolysis products of different plastic wastes, it was found that other substances, such as PP and PE, have negligible influences on the degradation of PET and the selectivity of BHET and even achieve 100% depolymerization of polyester products. (Figure 5G).

These results prove that Zn─O─Zn sites with similar structures to those of natural enzymes exhibit superior activity, selectivity, and energy efficiency, offering greater stability and scalability. However, due to different methods, hydrolysis and glycolysis eventually lead to different products. Hydrolysis is more conducive to the formation of terephthalic acid as a basic chemical raw material, whereas glycolysis is more convenient for plastic regeneration.

### 3.2 Nanozymes for Pollutant Degradation

With the rapid development of human society, many pollutants are discharged, including petrochemical products, pesticides and persistent organic pollutants. For the sake of human health, ecological balance and sustainable development of society, the core mission in the field of global environmental science and technology is to develop and apply efficient, economical and environment‐friendly pollutant degradation technologies. Among them, the use of emerging nanozymes, including peroxidase mimics, laccase mimics, and hydrolase mimics, to degrade pollutants has attracted extensive research interest.

#### 3.2.1 Peroxidase Mimics

**FIGURE 6:**
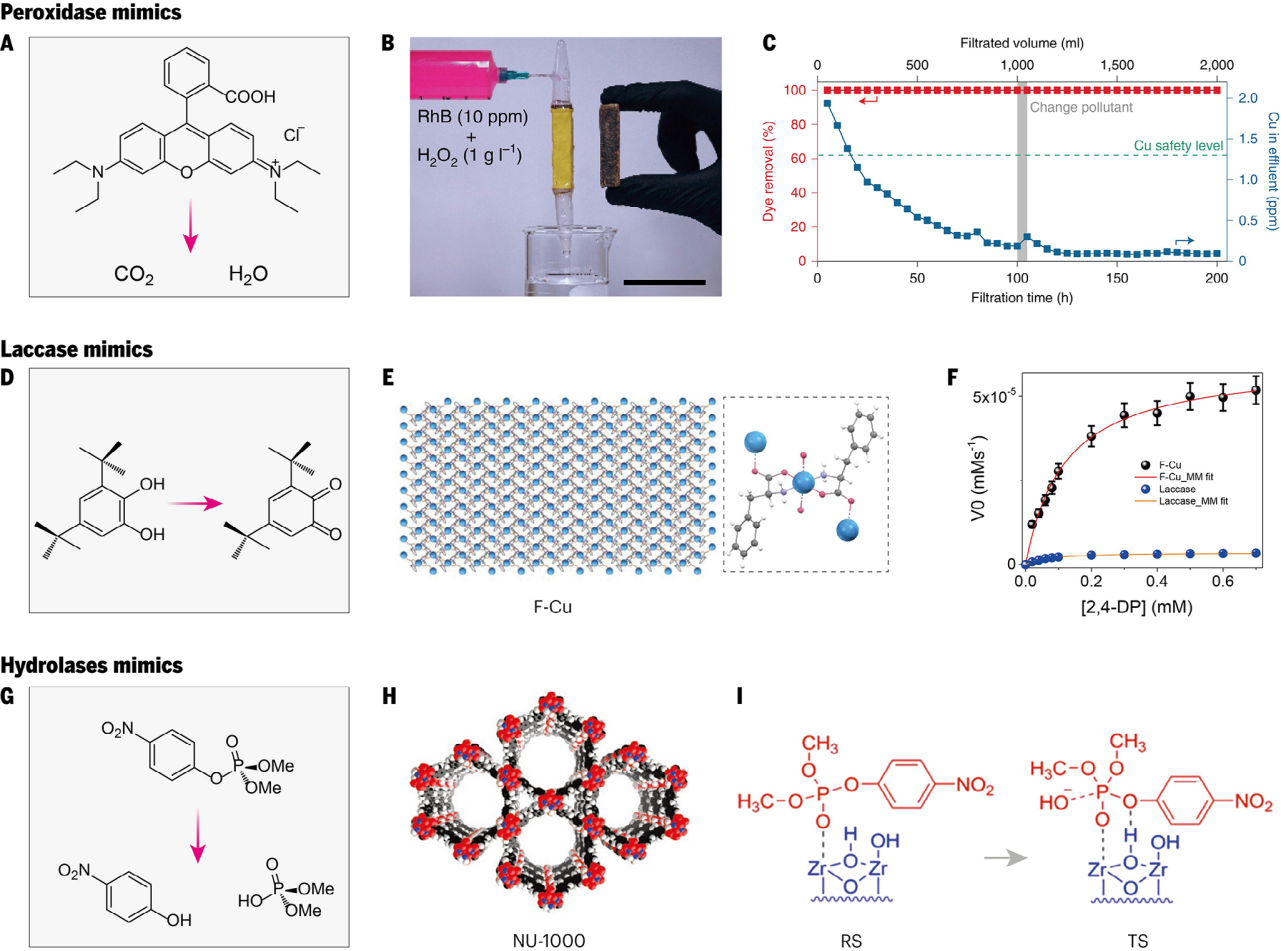
Nanozymes for pollutant degradation. A) Degradation of Rhodamine B (Rh B) catalyzed by a peroxidase mimic. B) Photograph of a proof‐of‐concept Fenton filter used for dye removal. C) Dye removal efficiency and Cu concentration in the effluent as a function of filtration time. B and C Reproduced with permission [65]. Copyright 2020, American Chemical Society. D) Oxidation of 3,5‐di‐tert‐butylcatechol (3,5‐DTBC) by a catechol oxidase mimic. E) 2D infinite layered structure of the F─Cu coordination complex and its octahedral primary coordination environment. F) Initial reaction rate (*V*₀) catalyzed by 0.1 mg mL^−1^ F–Cu or laccase as a function of substrate concentration. E and F Reproduced with permission [66]. Copyright 2022, The Authors. G) Catalytic decomposition of dimethyl 4‐nitrophenyl phosphate (DMNP) by hydrolase mimics. H) Molecular structure of the NU‐1000 metal–organic framework node and organic linker. I) Schematic illustration of the optimized reactant state (RS) and transition state (TS) structures during DMNP hydrolysis catalyzed by NU‐1000. H and I Reproduced with permission [67]. Copyright 2018, American Chemical Society.

The degradation of refractory organic pollutants by peroxidase mimics relies primarily on the generation of hydroxyl radicals (⚫OH), which are highly oxidative species that are central to advanced oxidation processes (AOPs). These reactive radicals exhibit nonselective reactivity toward organic substrates and can achieve complete mineralization of contaminants, exemplified by the conversion of rhodamine B (RhB) into CO_2_ and H_2_O (Figure 6A). Xu et al. reported the design of a copper single‐atom catalyst incorporated into graphitic carbon nitride (Cu‐C_3_N_4_), which activates H_2_O_2_ to generate ⚫OH efficiently under neutral pH conditions [65]. Neutrality is the pH of most practical wastewater, so compared with Fenton reagent, Cu─C_3_N_4_ avoids wasting a large amount acid to adjust the pH, and the heterogeneous catalyst can be recovered. The uniform dispersion of isolated Cu sites minimizes radical quenching and enhances stability, whereas the immobilization of the catalyst in a Fenton‐type filter circumvents recovery issues. This catalytic system achieved near‐complete mineralization of RhB in only 5 min (Figure 6B). The removal efficiency for RhB was significantly greater than that of conventional Cu‐containing catalysts such as Cu_2_O or CuO, and the catalyst maintained excellent performance over multiple cycles with minimal metal leaching (Figure 6C), adhering to environmental safety standards. Using the same H_2_O_2_ electrolyzer and Fenton filter, the synthetic wastewater containing three pollutants was completely oxidized by a Fe_3_O_4_‐carbon filter within 100 h, which confirmed the feasibility of the organic wastewater treatment system.

#### 3.2.2 Laccase Mimics

Laccase, a multicopper‐containing enzyme, has gained significant attention for its ability to oxidize toxic phenolic compounds without H_2_O_2_, facilitating their conversion into benign polymeric substances. By mimicking the structure and function of laccases, the instability of degrading pollutants in practical solutions can be improved. Li et al. strategically engineered MOF‐818 to mimic the trinuclear copper center found in natural catechol oxidases and effectively catalyzed the oxidation of 3,5‐di‐tert‐butylcatechol (3,5‐DTBC) to its corresponding o‐quinone in the presence of oxygen (Figure 6D), which proved the feasibility of structural bionics [68]. In addition, Makam et al. designed a hierarchical 2D layered bionanozyme (F‐Cu) utilizing the aromatic stacking effect of phenylalanine (F) and its amino/carboxyl coordination characteristics to strongly complex with redox‐active Cu^2+^ (Figure 6E) [66]. This structural simplicity not only minimizes synthetic complexity but also ensures exceptional robustness across variations in pH, ionic strength, and temperature. Based on the oxidation reaction between 2,4‐ dichlorophenol (2,4‐ DP) and 4‐aminoantipyrine (4‐AP), the enzyme‐like behavior of F─Cu was confirmed by Michaelis–Menten fitting (Figure 6F), which revealed a maximum velocity (*V*_max_ = 6 × 10^−5^ mm s^−1^) and a Michaelis−Menten constant(*K*_m_ = 0.19 mm), which far surpassed those of natural laccases (*V*_max_ = 3 × 10^−6^ mm s^−1^; *K*_m_ = 0.06 mm). In the whole reaction process, phenolic molecules are weakly coordinated with exposed Cu sites on the surface, in which the phenolic proton is temporarily stored on the carboxylate group of phenylalanine, generating phenoxyl radicals. The resulting phenoxyl radicals undergo spontaneous coupling to form low‐toxicity oligomeric or polymeric products. The Cu^2^^+^/Cu^+^ redox couple operates as an electron relay during this process, enabling continuous catalytic turnover. Besides, the activity in real water samples was compared. The results show that F‐Cu and laccase have similar activities in tap water and river water. However, in seawater, F─Cu maintains 76% activity and laccase is completely inactivated, which shows that F─Cu is stable enough. Moreover, according to the function bionics, single‐atom ruthenium [69] and SiO_2_@MnO_2_ [70] nanozymes catalyze the oxidation of substrates by producing reactive oxygen species, mimicking the catalytic function of laccase and realizing the recognition and degradation of catechol and resorcinol. It was observed that the imitations of laccase generally contain valence‐changing metals such as copper, cerium, and manganese, which is similar to copper in natural laccase, and it is a site where the electronic structure can be adjusted more easily.

#### 3.2.3 Hydrolase Mimics

Organophosphorus nerve agents represent a class of highly toxic chemicals that pose acute risks to human health [71]. In biological systems, their detoxification is mediated by phosphotriesterase, which catalyzes hydrolysis at a Lewis acidic Zn─OH─Zn center. Inspired by this enzymatic motif, Zr‐based metal–organic frameworks (Zr‐MOFs) featuring structurally analogous Zr─OH─Zr nodes have been explored as biomimetic catalysts capable of promoting organophosphorus hydrolysis [72]. To evaluate their catalytic activity, 4‐nitrophenyl phosphonate (DMNP) is typically employed as a low‐toxicity surrogate, whose hydrolysis yields p‐nitrophenol that can be quantitatively monitored by UV–vis spectroscopy (Figure 6G). Mondloch et al. reported a Zr‐MOF, NU‐1000, with eight connected Zr_6_ nodes exposing a high concentration of accessible Lewis‐acidic Zr sites (Figure 6H) [73]. Compared with UIO‐66, NU‐1000 possesses exceptionally large mesoporous channels (∼31 Å) that facilitate the unrestricted diffusion of bulky nerve agent molecules into the vast interior surface area. The experimental results demonstrated that NU‐1000 achieved a half‐life of 15 min for DMNP, significantly outperforming previously reported MOFs such as UiO‐66. Crucially, NU‐1000 also exhibited extraordinary activity against the highly toxic CWA Soman (GD), with a half‐life of only 3 min in a buffered solution and 36 min under 50% relative humidity, representing an 80‐fold enhancement over benchmark materials such as HKUST‐1. Notably, the thermal dehydration of Zr‐based MOFs generates coordinatively unsaturated Zr(IV) centers within Zr─OH─Zr nodes that closely emulate the active sites of phosphotriesterase enzymes. Chen et al. substantiated this biomimetic feature through density functional theory (DFT) calculations, which elucidated a cooperative hydrolysis mechanism [67]: one Zr center coordinates with the phosphate ester group to activate the phosphorus atom, whereas an adjacent hydroxyl group forms a hydrogen bond with the oxygen atom of the leaving group, thereby enhancing its departure (Figure 6I). Such cooperative interactions are analogous to enzymatic catalysis, wherein multiple site‐specific interactions stabilize the transition state and reduce the activation barrier.

### 3.3 Nanozymes for CO2 Capture

**FIGURE 7:**
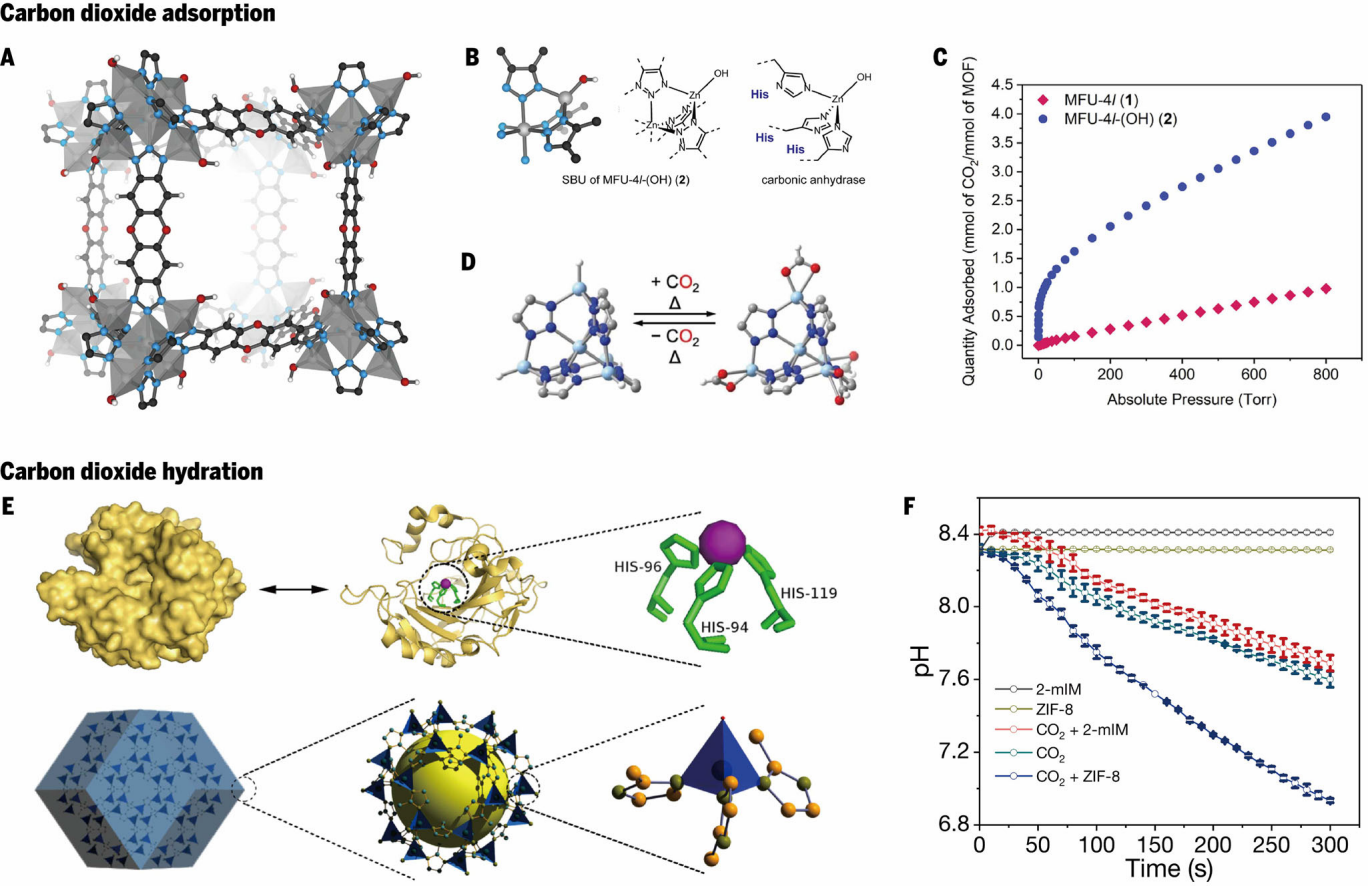
Nanozymes for CO_2_ capture. A) Crystal structure of MFU‐4*l* functionalized with hydroxyl groups (MFU‐4*l*‐(OH)). B) Structural comparison of the active site of human carbonic anhydrase II (hCAII) and the peripheral zinc site in MFU‐4*l*. C) CO_2_ adsorption isotherm of MFU‐4*l* measured at 298 K. A, B, and C Reproduced with permission [74]. Copyright 2018, Elsevier Inc. D) Reversible insertion of CO_2_ into Zn–H bonds in ZnHMFU‐4*l*. Reproduced with permission [75]. Copyright 2024, The Author. E) Structural comparison between hCAII (PDB ID: 1H9N) and the metal–organic framework ZIF‐8. F) The pH decay with continuous flow of CO_2_ gas. E and F Reproduced with permission [76]. Copyright 2019, Royal Society of Chemistry.

Strategies to combat global warming, in addition to the aforementioned carbon reduction methods, also include carbon capture, which is an important measure that not only reduces carbon emissions but also facilitates subsequent utilization. Metal–organic frameworks (MOFs) have great advantages in gas adsorption and storage because of their porous structure and large specific surface area [77]. Moreover, MOFs have also become an ideal platform for simulating metalloenzymes because of their diverse structural design and stability [78]. Wright et al. successfully obtained MFU‐4*l*‐(OH) by transforming the Zn─Cl site in MFU‐4*l* into Zn─OH site, and its crystal structure is shown in Figure 7A [74]. Figure 7B shows that the coordination environment of the secondary building unit N_3_Zn─OH of MFU‐4*l*‐(OH) is highly similar to that of the carbonic anhydrase (CA) active center. Various spectroscopic data (EDX, XPS, XAS) confirmed the successful incorporation of hydroxide ligands. The CO_2_ adsorption isotherm shows that MFU‐4*l*‐(OH) exhibited remarkable CO_2_ adsorption behavior (Figure 7C). At room temperature, CO_2_ inserts into the Zn–OH bonds, leading to exceptionally high uptake at low pressures and a total adsorption capacity of 3.41 mmol g^−1^, which is consistent with the Zn─OH number in the MOF. This chemisorption process is comparable to the CA mechanism, where CO_2_ hydration proceeds via bicarbonate formation. Diffuse reflectance infrared spectroscopy revealed characteristic vibrational bands corresponding to bicarbonate species, further confirming the analogy. Beyond adsorption, MFU‐4*l*‐(OH) displays catalytic reactivity that parallels that of CA, accelerating the oxygen isotope exchange between H_2_O and CO_2_ and catalyzing the hydrolysis of 4‐nitrophenyl acetate. Compared with MFU‐4*l* and other MOFs, MFU‐4*l*‐(OH) consistently demonstrates superior performance because of its preinstalled Zn─OH sites, which circumvent the need for in situ ligand exchange. Similarly, replacing Zn─Cl with Zn─H also results in the reversible insertion of CO_2_ at high temperatures, which is beneficial for the capture of industrial carbon dioxide (Figure 7D) [75].

The coordination environment of the metal center Zn and 2‐methylimidazole of ZIF‐8 is similar to the Zn(His)_3_O core of human carbonic anhydrase II (hCAII), enabling ZIF‐8 to perform enzyme‐like reactions (Figure 7E). Chen et al. demonstrated that surface Zn^2+^ ions with incomplete coordination act as Lewis acid sites, lowering the pK_a_ of coordinated water molecules and facilitating the formation of zinc‐bound hydroxides [76]. This feature endows ZIF‐8 with hydratase activity toward CO_2_, mimicking the natural process of CA that accelerates CO_2_ hydration into bicarbonate and protons. Control experiments revealed that the catalytic hydration of CO_2_ by ZIF‐8 significantly decreases the solution pH, highlighting its potential in CO_2_ capture and conversion strategies (Figure 7F). Beyond CO_2_ hydration, ZIF‐8 also efficiently catalyzes p‐nitrophenyl acetate (pNPA) hydrolysis to p‐nitrophenol, exhibiting Michaelis–Menten kinetics with comparable substrate affinity to hCAII. Moreover, ZIF‐8 is capable of catalyzing the hydrolysis of acetylthiocholine, confirming its versatility in mimicking multiple enzyme classes. Interestingly, the catalytic efficiency of ZIF‐8 is influenced by the particle size, with smaller nanoparticles exhibiting superior performance because of their higher surface defect density. The framework also displays high stability and recyclability, retaining activity after multiple catalytic cycles. These findings provide evidence that an MOF not only structurally mimics but also functionally reproduces an active enzyme. Notably, the Zn in r‐Zn–MOF74–NT mentioned earlier also has Lewis acidity and can break the ester bond. The hydrolase‐like activity of ZIF‐8 further proves the importance of functionalized Zn in ester hydrolysis.

## 4 Outlook

The rapid development of nanozyme research has created new opportunities for addressing pressing challenges in energy conversion and environmental sustainability. By combining the ability to mimic natural enzymatic functions with superior robustness, cost‐effectiveness, and structural tunability, nanozymes have emerged as a promising class of next‐generation catalysts. Nevertheless, despite notable progress, their translation into practical technologies remains at a relatively early stage. 1) A primary challenge concerns the incomplete understanding of nanozyme catalytic mechanisms at the atomic and molecular levels. In contrast to natural enzymes, where active sites and reaction pathways are well characterized, many nanozymes operate through complex and often ambiguous surface‐mediated processes. Establishing well‐defined structure–activity relationships through advanced in situ characterization techniques and rigorous theoretical modeling will be essential for enabling rational design. 2) Enhancing catalytic selectivity and substrate specificity represents another major hurdle, particularly in complex matrices. Future research should prioritize interface engineering, the introduction of site‐specific functionalities, and the exploitation of cooperative or tandem catalysis to approximate the complexity of multienzyme systems. The integration of machine learning with high‐throughput screening strategies may further accelerate the identification of nanozyme architectures with optimized performance. 3) From an application perspective, issues of long‐term operational stability, biocompatibility, and environmental safety require careful consideration. A comprehensive evaluation of nanozyme degradation pathways, leaching behavior, and ecological impact is critical before deployment in environmental or biomedical contexts can be realized. Moreover, the establishment of scalable synthesis routes that ensure reproducibility, uniformity, and cost efficiency remains indispensable for advancing toward industrial implementation. 4) The convergence of nanozyme science with synthetic biology, soft robotics, and smart materials holds the potential to enable adaptive, responsive, and self‐regulating catalytic systems. In energy applications, the incorporation of nanozymes into integrated electrochemical devices and hybrid catalytic networks could increase both the efficiency and functional versatility. In environmental remediation, modular and field‐deployable nanozyme platforms may provide decentralized solutions for pollutant treatment in remote or resource‐limited regions.

In conclusion, nanozymes represent a highly versatile platform for sustainable catalysis. Realizing their full potential will require sustained interdisciplinary efforts that bridge fundamental mechanistic understanding with application‐oriented innovation, underpinned by advances in materials chemistry, bioengineering, and environmental science.

## Conflicts of Interest

The authors declare no conflict of interest.

## Data Availability

The data that support the findings of this study are available in the supplementary material of this article.
